# Enteric glial NLRP3 inflammasome contributes to gut mucosal barrier alterations in a mouse model of diet‐induced obesity

**DOI:** 10.1111/apha.14232

**Published:** 2024-09-17

**Authors:** Vanessa D'Antongiovanni, Matteo Fornai, Rocchina Colucci, Anna Nericcio, Laura Benvenuti, Clelia Di Salvo, Cristina Segnani, Clarissa Pierucci, Chiara Ippolito, Zoltan H. Nemeth, György Haskó, Nunzia Bernardini, Luca Antonioli, Carolina Pellegrini

**Affiliations:** ^1^ Unit of Histology and Medical Embryology, Department of Clinical and Experimental Medicine University of Pisa Pisa Italy; ^2^ Unit of Pharmacology and Pharmacovigilance, Department of Clinical and Experimental Medicine University of Pisa Pisa Italy; ^3^ Department of Pharmaceutical and Pharmacological Sciences University of Padova Padova Italy; ^4^ Department of Anesthesiology Columbia University Irving Medical Center New York New York USA; ^5^ Department of Surgery Morristown Medical Center Morristown New Jersey USA

**Keywords:** enteric glia, high fat‐diet, inflammasome, intestinal epithelial barrier, intestinal inflammation, mucosal inflammation, obesity

## Abstract

**Aim:**

In the present study, we investigated the involvement of NLRP3 inflammasome in the intestinal epithelial barrier (IEB) changes associated with obesity, and its role in the interplay between enteric glia and intestinal epithelial cells (IECs).

**Methods:**

Wild‐type C57BL/6J and NLRP3‐KO (^−/−^) mice were fed with high‐fat diet (HFD) or standard diet for 8 weeks. Colonic IEB integrity and inflammasome activation were assessed. Immunolocalization of colonic mucosal GFAP‐ and NLRP3‐positive cells along with in vitro coculture experiments with enteric glial cells (EGCs) and IECs allowed to investigate the potential link between altered IEB, enteric gliosis, and NLRP3 activation.

**Results:**

HFD mice showed increased body weight, altered IEB integrity, increased GFAP‐positive glial cells, and NLRP3 inflammasome hyperactivation. HFD‐NLRP3^−/−^ mice showed a lower increase in body weight, an improvement in IEB integrity and an absence of enteric gliosis. Coculture experiments showed that palmitate and lipopolysaccharide contribute to IEB damage and promote enteric gliosis with consequent hyperactivation of enteric glial NLRP3/caspase‐1/IL‐1β signaling. Enteric glial‐derived IL‐1β release exacerbates the IEB alterations. Such an effect was abrogated upon incubation with anakinra (IL‐1β receptor antagonist) and with conditioned medium derived from silenced‐NLRP3 glial cells.

**Conclusion:**

HFD intake elicits mucosal enteric gliotic processes characterized by a hyperactivation of NLRP3/caspase‐1/IL‐1β signaling pathway, that contributes to further exacerbate the disruption of intestinal mucosal barrier integrity. However, we cannot rule out the contribution of NLRP3 inflammasome activation from other cells, such as immune cells, in IEB alterations associated with obesity. Overall, our results suggest that enteric glial NLRP3 inflammasome might represent an interesting molecular target for the development of novel pharmacological approaches aimed at managing the enteric inflammation and intestinal mucosal dysfunctions associated with obesity.

## 1 INTRODUCTION

Obesity is a multifactorial disease strongly linked to several medical comorbidities, such as type‐2 diabetes mellitus, cardiovascular diseases, gastrointestinal (GI) disorders, and cognitive impairment.^1^, ^2^, ^3^ In the recent years, increasing efforts have been addressed to identify a common root underlying obesity and related comorbidities, pointing out the alterations of intestinal mucosal barrier as a common pathological feature.^4^, ^5^, ^6^

Compelling evidence well demonstrated that high fat‐diet (HFD) intake, besides to induce changes in gut microbiota composition, determinates alterations of mucosal barrier integrity, thus facilitating the translocation of luminal noxious molecules. The influx of luminal antigens activate the mucosal immune system thus eliciting the onset of a low‐grade systemic inflammation.^5^, ^6^ The continuous release of pro‐inflammatory cytokines undermines the intestinal barrier integrity, thus amplifying immune activation and inflammatory reactions.^7^, ^8^ The chronicization of such phlogistic condition alters the homeostatic mechanisms, thus leading to the development of different obesity‐associated comorbidities.^9^

Under physiological condition, the integrity of intestinal mucosal barrier results from a balanced interplay between gut microbiota, intestinal epithelial cells (IECs) and immune cells, and the enteric nervous system (ENS).^2^ In this context, enteric glial cells (EGCs), acting as an ideal bridge between the immune/inflammatory cells and the enteric neurons, modulate several physiologic gut activities, including immune response and barrier function.^10^, ^11^ Accordingly, morphofunctional alterations of enteric glia in inflammatory processes, also designed as reactive gliosis, contribute critically to their maintenance as well as to the impairment of intestinal mucosal barrier integrity and function.^12^, ^13^, ^14^

In last years, the nucleotide‐binding oligomerization domain leucine rich repeat and pyrin domain containing protein 3 (NLRP3) inflammasome multiprotein complex, expressed in immune/inflammatory cells and EGCs, is emerging as critical player in the pathogenesis of inflammatory responses associated with obesity via the processing and release of interleukin (IL)‐1β and IL‐18.^15^, ^16^, ^17^ Preclinical and clinical studies reported an hyperactivation of NLRP3 inflammasome signaling pathway in the presence of obesity, characterized by an increase in caspase‐1 activity and IL‐1β levels, in immune cells infiltrating the adipose tissue, thus corroborating the involvement of NLRP3 inflammasome in the meta‐inflammation associated with obesity.^15^, ^17^ Recently, Kimono et al., displayed that the activation of NLRP3 inflammasome in EGCs elicited by lipopolysaccharide (LPS) triggers a damage in intestinal epithelial barrier (IEB), highlighting a putative contribution of glial NLRP3 inflammasome in the pathophysiology of intestinal barrier disruption in vitro.^16^ However, the role of enteric glial NLRP3 inflammasome in the pathogenesis of intestinal mucosal barrier alterations associated with obesity remain still unclear.

Based on these premises, the present research work was focused on investigating the involvement of NLRP3 inflammasome in the mechanisms underlying the alterations of the intestinal mucosal barrier associated with obesity, focusing the attention on its role in the interplay between enteric glia and IECs.

## 2 RESULTS

### 2.1 In vivo experiments

#### 2.1.1 HFD NLRP3 KO (NLRP3 ^−/−^) mice developed lower increase in body weight

**FIGURE 1:**
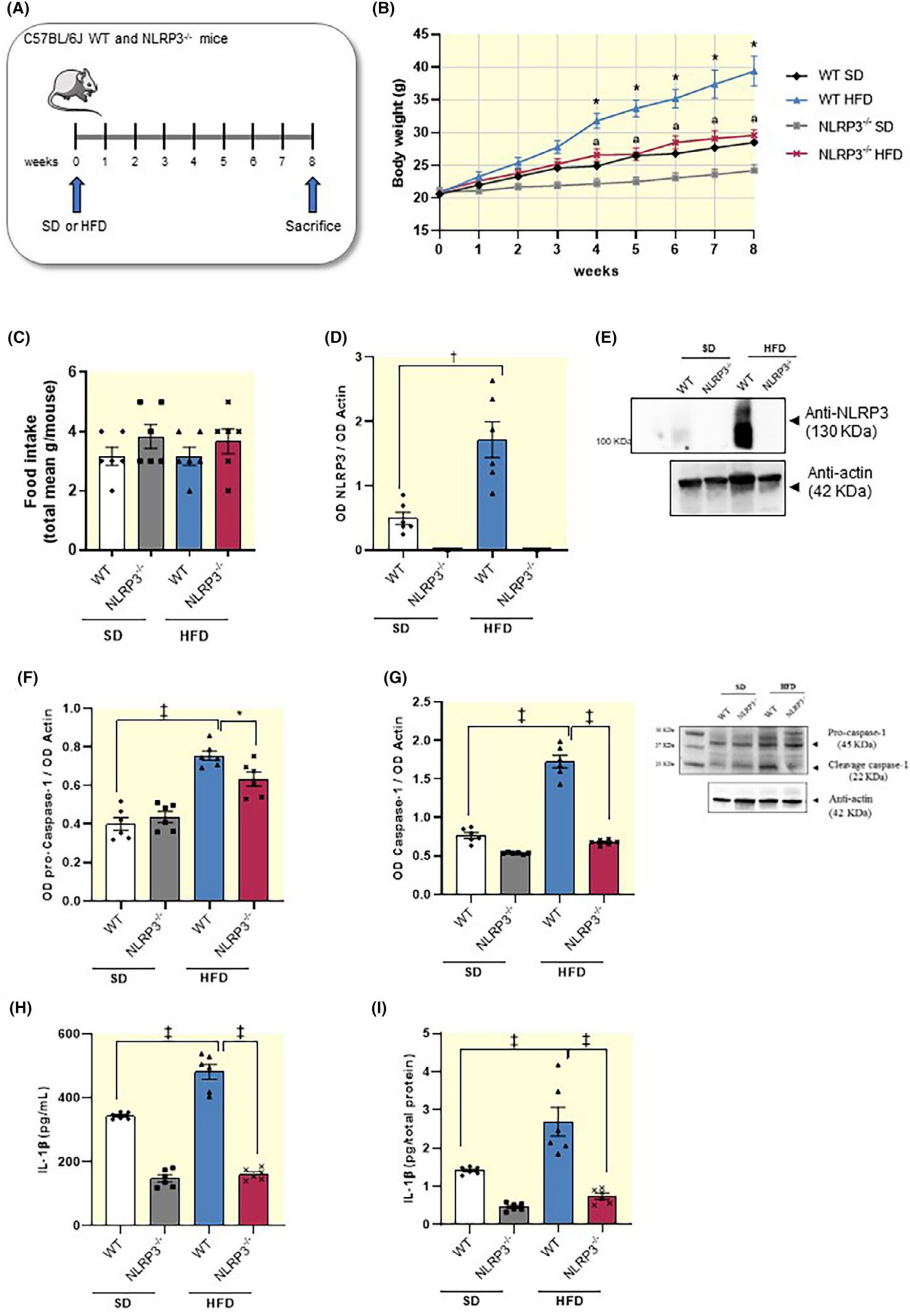
HFD‐NLRP3^−/−^ mice showed a lower increase in body weight and a normalization of inflammatory parameters. (A) Schematic representation of experimental design on WT or NLRP3^−/−^ mice fed with SD or HFD for 8 weeks. (B) Body weight (g) in WT or NLRP3^−/−^ mice fed with SD or HFD (*n* = 6/group). Two‐way ANOVA followed by Tukey's post hoc test results *p < 0.05, significant difference versus SD‐WT; ^a^ *p* <0.05, significant difference versus HFD‐WT. (C) Food intake in WT or NLRP3^−/−^ mice fed with SD or HFD. Data are means ± S.E.M (*n* = 6/group). (D) A scatter plot representing the densitometric analysis and (E) related representative blot of NLRP3 expression in colonic tissues from WT or NLRP3^−/−^ mice fed with SD or HFD. (F, G) A scatter plot representing the densitometric analysis and related representative blot of pro‐caspase‐1 and caspase‐1 expression assessed by Western blot analysis in colonic tissues from WT or NLRP3^−/−^ mice fed with SD or HFD. (H) A scatter plot representing the plasmatic and (I) colonic IL‐1β levels in WT or NLRP3^−/−^ mice fed with SD or HFD. Dots show values per individual mouse (*n* = 6/group) whereas black bars indicate means ± SEM. *p < 0.05, †*p* < 0.01 and ‡*p* < 0.001. One‐way ANOVA. HFD, high fat diet; NLRP3^−/−^, NLRP3 knockout mice; SD, standard diet; WT, wild‐type mice.

To corroborate the role of NLRP3 inflammasome in the onset of HFD‐induced obesity,^17^ we performed experiments in mice with NLRP3 gene deletion (Figure 1A). Standard diet (SD)‐NLRP3 KO (NLRP3^−/−^) mice displayed a slight decrease in body weight, although not significantly, as compared with SD‐wild‐type (WT) mice (Figure 1B). HFD‐WT mice showed a significant increase in body weight, as compared with SD‐WT mice (Figure 1B). In HFD‐NLRP3^−/−^ mice, the body weight gain was significantly lower as compared with HFD‐WT animals (Figure 1B). Overall, these results suggest that NLRP3^−/−^ mice were less susceptible to the development of HFD‐induced obesity, thus corroborating the relevant role of NLRP3 inflammasome in the pathophysiology of obesity. Of note, no significant differences in food intake were detected in SD and HFD WT or NLRP3^−/−^ mice (Figure 1C).

#### 2.1.2 HFD‐NLRP3 ^−/−^ mice showed a normalization of NLRP3 inflammasome‐related inflammatory parameters

Obesity is associated with a low‐grade systemic inflammation, that seems to contribute to the intestinal mucosal barrier dysfunctions associated with a hypercaloric diet.^4^, ^7^, ^8^ In this context, NLRP3 inflammasome is emerging as critical player in the pathogenesis of inflammatory responses associated with obesity.^15^, ^17^ Consistently, we found that HFD‐WT mice displayed a significant increase in the expression levels of colonic NLRP3 inflammasome subunit, pro‐caspase‐1 and cleaved caspase‐1 (p20, an autoprocessed fragment of caspase‐1) (Figure 1D–G). In parallel, it has been observed an increase in plasma and colonic IL‐1β levels, as compared with SD‐WT animals (Figure 1H,I), thus indicating the activation of the well‐established pattern of canonical NLRP3/caspase‐1/IL‐1β inflammasome signaling pathway. NLRP3 subunit was not expressed in NLRP3 deficient mice fed with SD and HFD (Figure 1E). In addition, NLRP3 gene deletion in SD and HFD mice was associated with a significant reduction in activated caspase‐1 expression along with a decrease in circulating and tissue IL‐1β levels, as compared with WT SD and HFD mice, respectively (Figure 1F–I). Taken together, these findings highlight that HFD‐NLRP3^−/−^ mice showed a normalization of NLRP3 inflammasome‐related inflammatory parameters, thus corroborating the relevant contribution of NLRP3 inflammasome in the onset and maintenance of enteric inflammation associated with obesity, via IL‐1β release.

#### 2.1.3 HFD NLRP3 ^−/−^ mice are characterized by the absence of enteric gliosis

**FIGURE 2:**
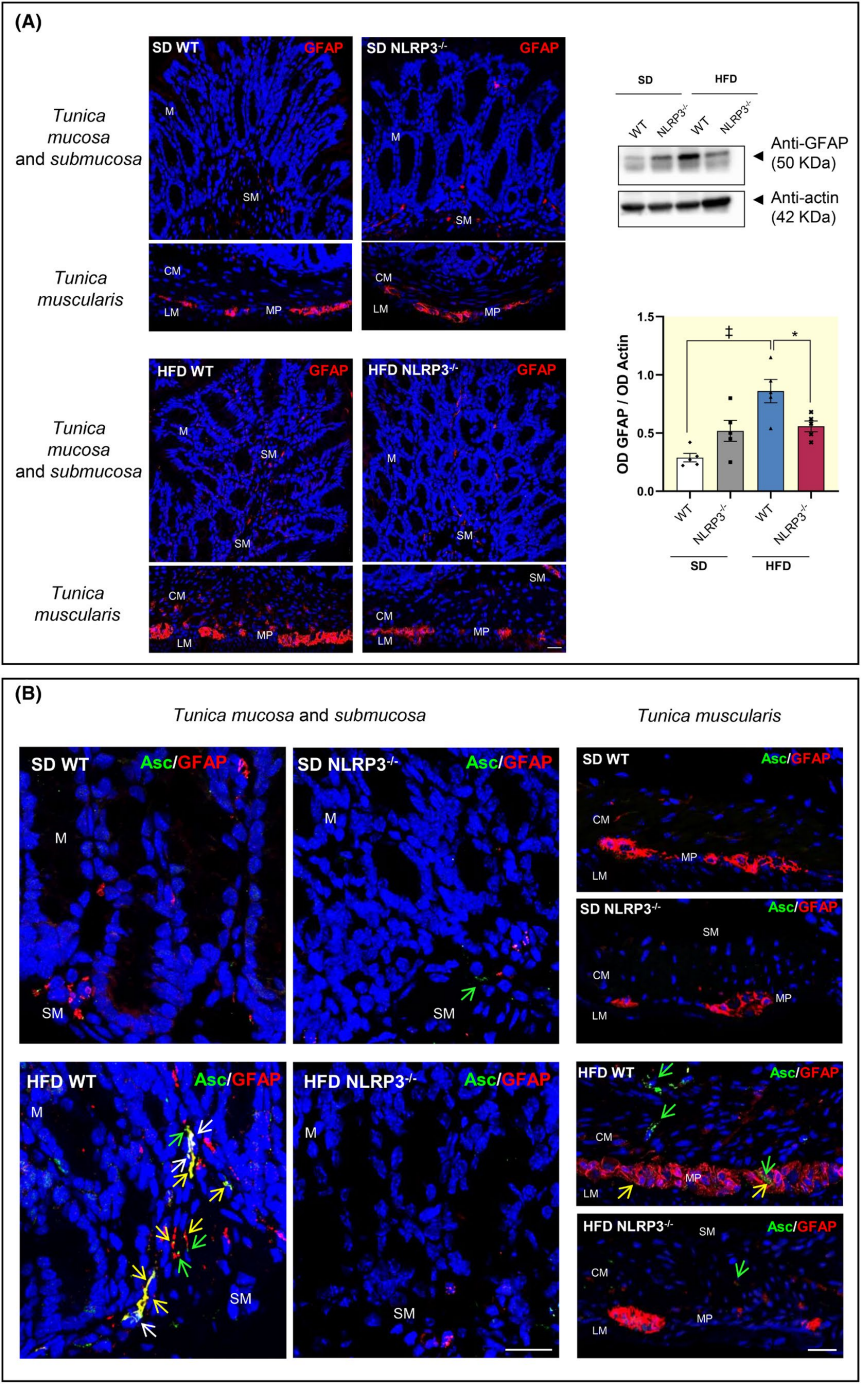
HFD‐WT mice displayed enteric glial NLRP3 activation. (A) Left: Representative confocal microscopy images of GFAP immunostaining of full‐thickness (*tunica mucosa/submucosa* and *tunica muscularis*), cross‐sectioned colon obtained from WT or NLRP3^−/−^ mice fed with SD or HFD. Scale bar: 20 μm. Right: A scatter plot representing the densitometric analysis and related representative blot of GFAP in colonic tissues from WT or NLRP3^−/−^ mice fed with SD or HFD. Dots show values per individual mouse (*n* = 5/group) whereas black bars indicate means ± SEM. *p < 0.05 and ‡*p* < 0.001. One‐way ANOVA. (B) Representative images of double immunofluorescence confocal microscopy of ASC (green) and GFAP (red) in mouse colon tissue sections. The GFAP‐positive glial cells in SD‐WT mice (left panel) are increased in HFD‐WT colon (right panel) and most of them display a granular ASC positivity widely colocalized with cytoplasmic GFAP^+^ (yellow fluorescence, yellow arrows) and nuclei (white fluorescence, white arrows). Scale bar: 20 μm. ASC, adaptor protein apoptosis‐associated speck‐like protein containing a caspase‐recruitment domain; CM, circular muscle layer; GFAP, glial fibrillary acidic protein; HFD, high fat diet; LM, longitudinal muscle layer; M, *tunica mucosa*; MP, myenteric plexus; NLRP3^−/−^, NLRP3 knockout mice; SD, standard diet; SM, *tunica submucosa*; WT, wild‐type mice.

Several studies have reported that, in the setting of obesity, EGCs shift toward a pro‐inflammatory phenotype, thus contributing relevantly to the initiation and maintenance of enteric inflammation.^15^, ^16^, ^17^ In the present study, we observed an increase in the colonic expression of glial marker, GFAP, along with an increase in GFAP‐positive glial cells in colonic *tunica mucosa* as well as in *tunica submucosa* and *muscularis* of HFD‐WT mice, as compared with SD‐WT mice (Figure 2A), suggesting the presence of reactive gliotic processes. Of interest, HFD‐NLRP3^−/−^ mice showed no increase in GFAP expression compared to the respective SD‐NLRP3^−/−^ mice, as well as to HFD‐WT mice. In addition, these mice showed a reduction of GFAP staining in *tunica mucosa* and *submucosa* as well as in myenteric plexus similar to that observed in SD mice WT and NLRP3^−/−^, indicating the absence of enteric gliosis (Figure 2A). No changes were observed in SD mice WT and NLRP3^−/−^ (Figure 2A).

#### 2.1.4 Colocalization between NLRP3 inflammasome and activated EGCs in colonic wall

Enteric glia includes several EGC subtypes, based on the morphology and localization within gut wall.^18^ Among them, EGCs located in the *tunica mucosa* exert a pivotal role in the regulation of IEB integrity and functions.^11^ Accordingly, to confirm the activation of the inflammasome complex in the intestinal activated EGCs, we performed a double‐staining immunofluorescence assay with ASC (marker for NLRP3 inflammasome complex) and GFAP (glial marker for EGCs) in colon tissue slices from SD and HFD WT and NLRP3^−/−^. Of note, ASC protein can activate the NLRP3 inflammasome only after the transition from a soluble state into an insoluble speck‐like state.^19^ Accordingly, we carried out the immunofluorescence assay with an ASC antibody able to detect ASC specks.^20^ Upon inflammasome assembly, ASC within the complex can be readily visualized inside cells, including intestinal epithelial cells and immune/inflammatory cells (i.e., monocytes, macrophages, dendritic cells, and T cells) by its oligomerization and the appearance of large aggregates, designated as speck.^21^ Double immunofluorescence experiments showed an abundant ASC‐positive specks in *tunica mucosa* as well as in *tunica submucosa* and *muscularis* of HFD‐WT mice, as compared with SD WT and NLRP3^−/−^ (Figure 2B), indicating the activation of inflammasome pathway. By contrast, HFD‐NLRP3^−/−^ showed an amount of ASC‐positive specks similar to that observed in SD WT and NLRP3^−/−^ mice, thus suggesting the absence of NLRP3 inflammasome activation. In addition, an abundant increase in the number of GFAP‐positive glial cells was observed in the colonic *tunica mucosa* as well as in *tunica submucosa* and *muscularis* from HFD‐WT mice, with more elongated processes, as compared with glial cells from SD WT and NLRP3^−/−^ mice (Figure 2B), corroborating the presence of reactive gliosis in obese mice. In this context, a number of GFAP‐positive cells showed ASC‐positive specks both in the nuclei and along their cytoplasmic processes, suggesting the activation of inflammasome pathways in the activated glial cells in the colonic wall of HFD‐WT mice (Figure 2B). Of note, we also observed some ASC‐positive speaks in other cell types, such as macrophages, in the *tunica muscularis* of HFD‐WT mice, in accordance with previous evidence.^17^ Overall, these results support the link between obesity, NLRP3 inflammasome activation and reactive gliosis at the level of the intestinal mucosa.

#### 2.1.5 Link among NLRP3 inflammasome activation, reactive gliosis and mucosal barrier alterations

In order to verify the existence of a link among NLRP3 inflammasome activation, enteric reactive gliosis and intestinal mucosal barrier alteration associated with obesity, we investigated the mucus layer composition, the expression of tight junction proteins (proteins involved in the maintenance of mucosal barrier integrity) and ZO‐1 localization in colonic tissues from SD‐ and HFD‐WT and NLRP3^−/−^ mice.

**FIGURE 3:**
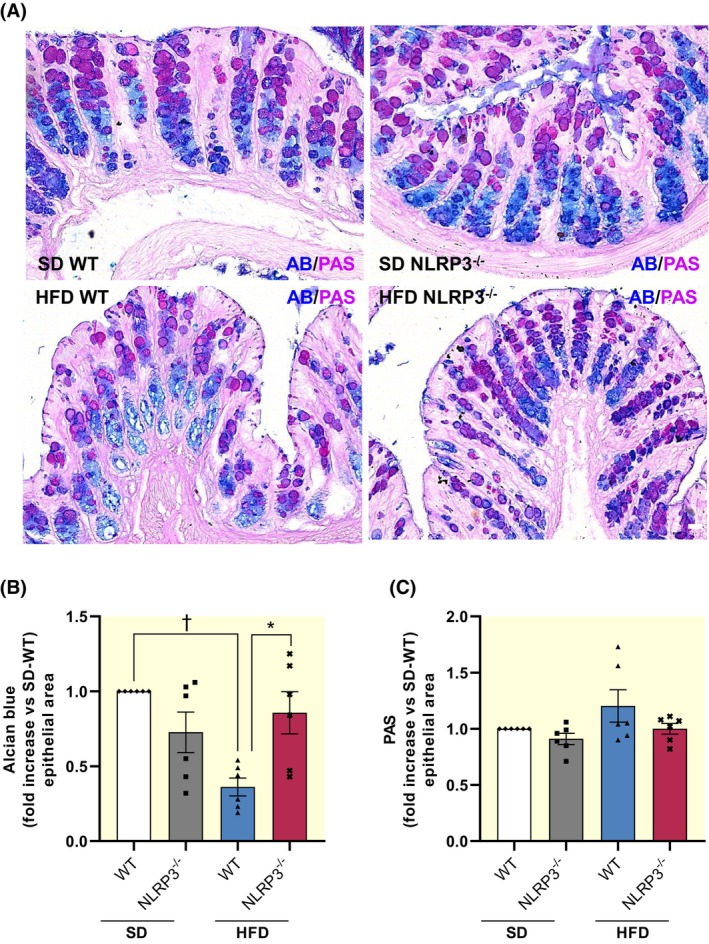
HFD‐NLRP3^−/−^ mice are not characterized by epithelial mucin disarrangement. (A) Representative images and (B, C) quantitative analysis of acidic (Alcian Blu, AB) and neutral (periodic‐acid‐Schiff's reaction, PAS) mucins in colonic tissues from WT or NLRP3^−/−^ mice fed with SD or HFD. Scale bar: 20 μm. Dots show values per individual mouse (*n* = 6/group) of fold changes of positive pixels percentages (PPP) of AB and PAS within the mucosal area examined, expressed as mean ± SEM. *n* = 6 for each experimental group; 3 sections/sample; 5 randomly selected fields/section. *p < 0.05 and †*p* < 0.01. One‐way ANOVA. HFD, high fat diet; NLRP3^−/−^, NLRP3 knockout mice; SD, standard diet; WT, wild‐type mice.

To investigate the mucus composition, we performed PAS and AB staining on colonic samples to quantify neutral and acidic mucins in the mucosal tubular glands. Of note, an altered balance of neutral/acidic mucin ratio has been reported in the presence of obesity, resulting in a decreased protective function of the mucus layer against pathogen translocation and an altered repair mechanism of the epithelium.^22^, ^23^ HFD‐WT mice showed a significant decrease in acidic mucins along with a rebalance of neutral ones, indicating an altered mucus production (Figure 3A–C). Interestingly, HFD‐NLRP3^−/−^ mice showed neutral and acidic mucins expression similar to those observed in SD‐WT mice, suggesting that the mucus secretion maintains its physiological composition in this strain (Figure 3A–C).

**FIGURE 4:**
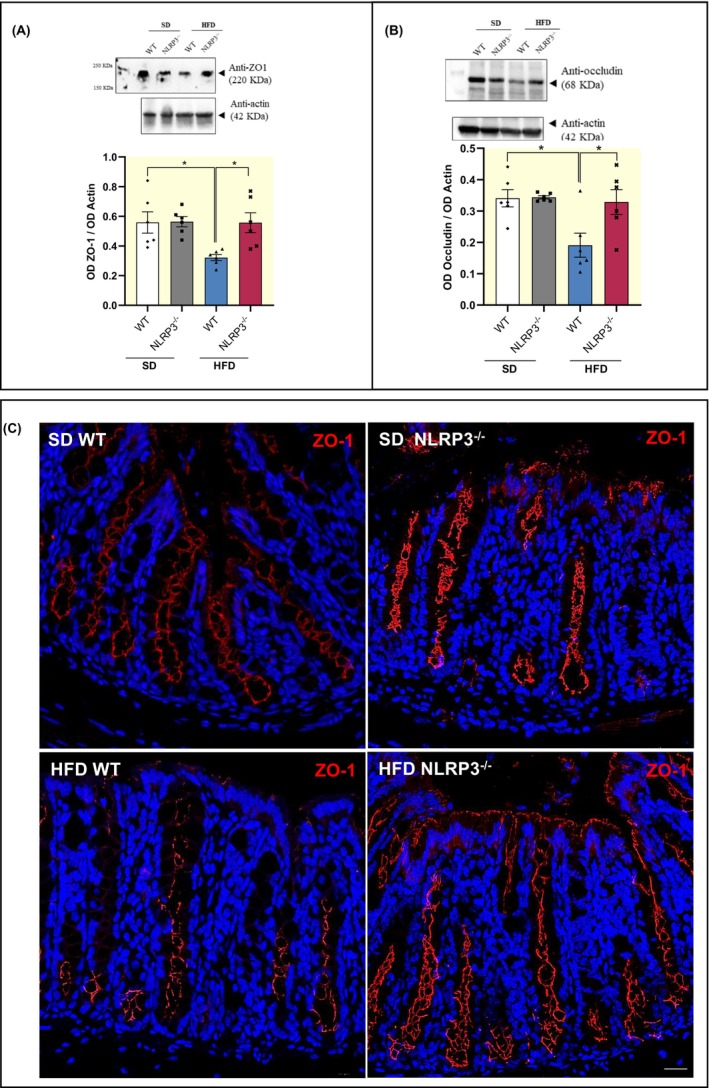
HFD‐NLRP3^−/−^ mice are characterized by a normalization of intestinal barrier integrity. (A, B) A scatter plot representing the densitometric analysis and related representative blot of ZO‐1 and occludin in colonic tissues from WT or NLRP3^−/−^ mice fed with SD or HFD. Dots show values per individual mouse (*n* = 6/group) whereas black bars indicate means ± SEM. *p < 0.05. One‐way ANOVA (C) Representative confocal microscopy images of ZO‐1 immunostaining of full‐thickness, cross‐sectioned colon obtained from WT or NLRP3^−/−^ mice fed with SD or HFD. Scale bar: 20 μm. HFD, high fat diet; NLRP3^−/−^, NLRP3 knockout mice; SD, standard diet; WT, wild‐type mice; ZO‐1, zonulin‐1.

In addition, HFD‐WT mice displayed a significant reduction in the colonic expression of zonulin‐1 (ZO‐1) and occludin, as compared with SD mice WT and NLRP3^−/−^ (Figure 4A,B). Of interest, HFD‐NLRP3^−/−^ mice were not associated with changes in ZO‐1 and occludin expression, as compared with HFD‐WT mice (Figure 4A,B), indicating that NLRP3^−/−^ mice (characterized by the absence of both NLRP3 inflammasome and mucosal enteric gliotic processes) are less susceptible to the development of mucosal barrier alterations associated with obesity. Of note, this evidence was corroborated with the immunofluorescence analysis of ZO‐1 showing an irregular and blurry signal in HFD‐WT mice, as compared with SD‐WT mice (Figure 4C). By contrast, HFD‐NLRP3^−/−^ mice showed a clear and sharp ZO‐1 staining in specific cellular sites like the cell boundaries, indicating that the structure of TJ maintains its physiological morphology in comparison with SD‐NLRP3^−/−^ mice (Figure 4C).

### 2.2 In vitro experiments

In order to investigate the potential relationship between NLRP3 inflammasome activation, enteric gliosis, and alterations of intestinal mucosal barrier in the setting of obesity, we performed in vitro experiments on coculture of EGCs and intestinal epithelial cells (IEC‐6 cells) treated with palmitate (PA) and lipopolysaccharide (LPS), to mimic the in vivo features of HFD exposure.

#### 2.2.1 Incubation with PA and LPS induces an increase in IEC‐6 trans‐epithelial permeability

As first step, we analyzed the expression levels of tight junction proteins and FITC‐dextran flux in IEC‐6 cells treated with PA and LPS directly in the medium in order to evaluate the effects of PA and LPS on the intestinal mucosal barrier integrity.

**FIGURE 5:**
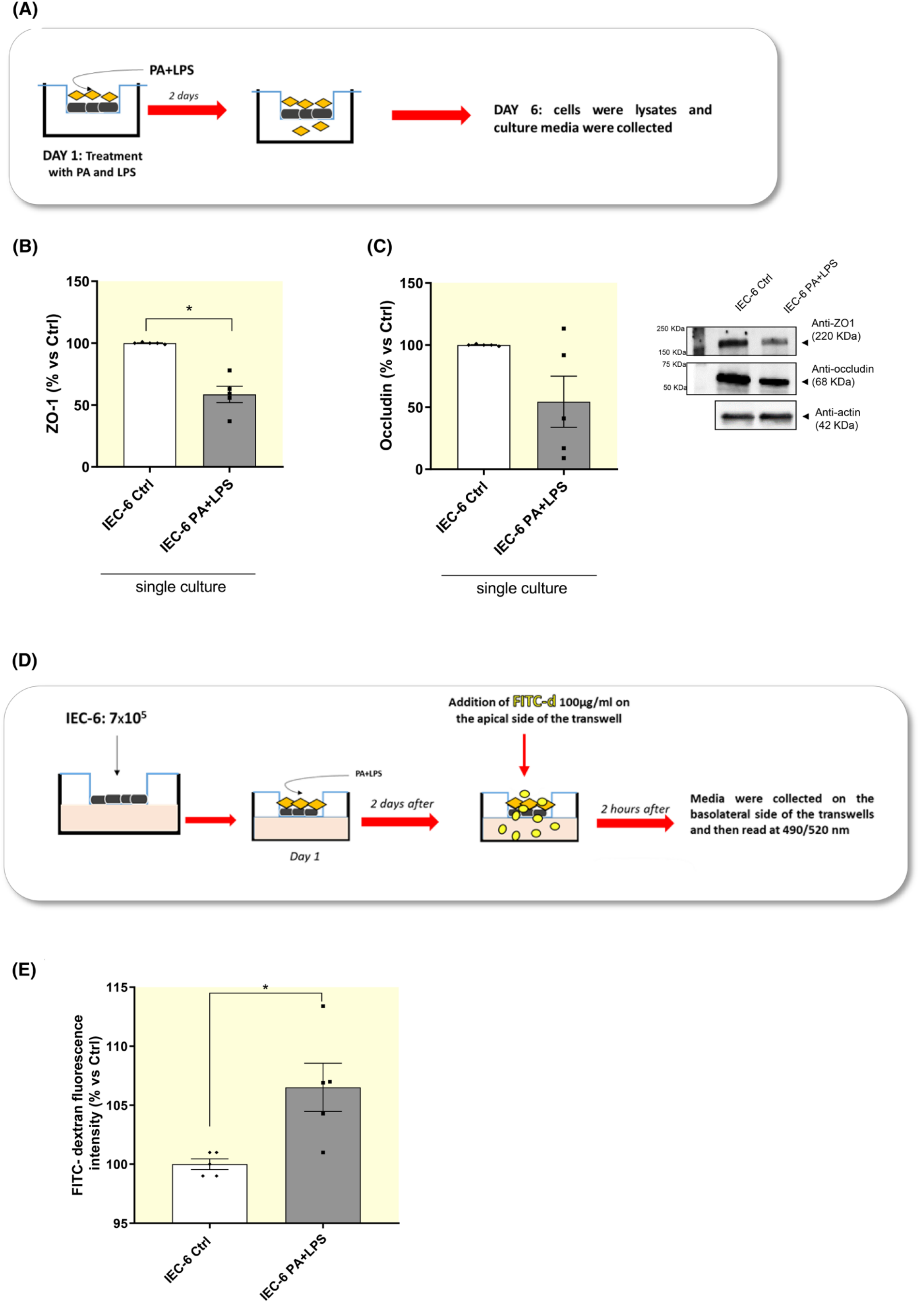
Incubation of PA and LPS increases trans‐epithelial permeability and induces enteric gliosis. (A) Schematic representation of culture experiment on IEC‐6 cells. Intestinal epithelial cells were treated with PA and LPS for 5 days. At day 6, cells were lysed and culture media were collected. (B, C) A scatter plot representing the densitometric analysis and related representative blot of ZO‐1 and occludin assessed by Western blot assay in IEC‐6 cells treated with PA and LPS. (D) Schematic representation of FITC‐dextran experiment on IEC‐6 cells. For experimental protocol details see “Section 4” and Figure 1B. (E) FITC‐dextran fluorescence intensity measured in IEC‐6 cells treated with PA and LPS. Dots show values per individual experiments (*n* = 5–6 independent experiments performed in duplicate) whereas black bars indicate means ± SEM. *p < 0.05. One‐way ANOVA. EGC, enteric glial cells; GFAP, glial fibrillary acidic protein; IEC‐6, intestinal epithelial cells; LPS, lipopolysaccharide; PA, palmitate; ZO‐1, zonulin‐1.

Treatment of IEC‐6 cells with PA and LPS determined a significant decrease in the expression of ZO‐1, whereas occludin expression was not significantly affected (Figure 5A–C). In addition, incubation with PA and LPS induced an increase in FITC‐dextran fluorescence in the lower compartment of trans‐well insert, thus indicating an increase in trans‐epithelial permeability (Figure 5D,E). Overall, these results suggest that a condition mimicking HFD exposure induces alterations in intestinal barrier integrity and permeability.

#### 2.2.2 PA and LPS promote enteric gliosis and NLRP3 inflammasome signaling activation

**FIGURE 6:**
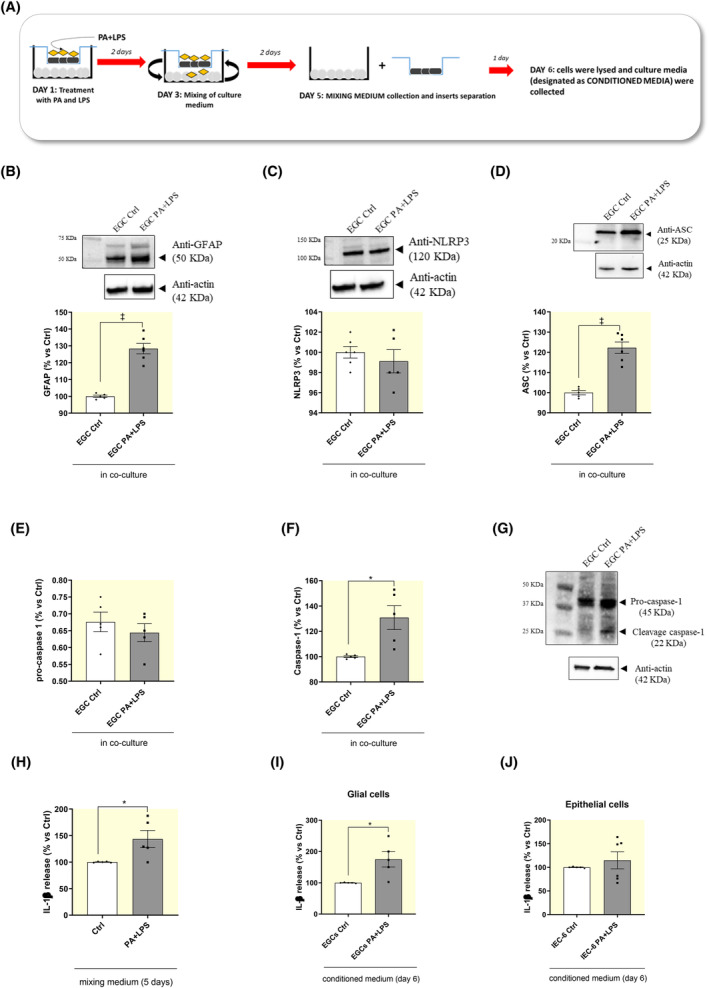
Incubation of PA and LPS triggers NLRP3 inflammasome activation in activated EGCs. (A) Schematic representation of coculture experiments. For experimental protocol details see “Section 4” and Figure 1A. (B) A scatter plot representing the densitometric analysis and related representative blot of GFAP in cocultured EGCs treated with PA and LPS. (C–G) A scatter plot representing the densitometric analysis and related representative blot of (C) NLRP3, (D) ASC, (E–G) pro‐caspase and (F, G) caspase‐1 in cocultured EGCs treated with PA and LPS. (H–J) A scatter plot representing the IL‐1β levels in the supernatants of (H) mixing medium, (I) conditioned medium derived from cocultured EGCs and (J) conditioned medium derived from cocultured IEC‐6 cells. Dots show values per individual experiments (*n* = 5–6 independent experiments performed in duplicate) whereas black bars indicate means ± SEM. *p < 0.05 and ‡*p* < 0.001. One‐way ANOVA. EGC, enteric glial cells; IEC‐6, intestinal epithelial cells; IL‐1β, interleukin‐1β; LPS, lipopolysaccharide; NLRP3, nucleotide‐binding oligomerization domain leucine rich repeat and pyrin domain containing protein 3; PA, palmitate.

As a consequence of the impairment in IEC‐6 monolayer integrity, soluble molecules contained in cell culture insert, such as LPS, are able to cross the porous membrane of trans‐well insert, going into the lower compartment, as demonstrated by an increased FITC‐dextran fluorescence. As a result, cocultured EGCs treated with PA and LPS showed a significant increase in the expression of glial marker GFAP, thus indicating enteric glial hyperactivation (Figure 6A,B). In GFAP‐positive glial cells no significant differences in the expression levels of NLRP3 inflammasome subunit and pro‐caspase‐1 were observed, while a significant increase in the expression levels of ASC inflammasome subunit and caspase‐1 were detected, as compared with cocultured control EGCs (Figure 6C–G), thus confirming that, in the setting of obesity, activated EGCs were characterized by an hyperactivation of canonical NLRP3/caspase‐1 inflammasome signaling pathway.

#### 2.2.3 GFAP‐positive enteric glial cells release high levels of IL‐1β via NLRP3 inflammasome activation

As anticipated above, NLRP3 inflammasome exerts a pivotal role in the pathogenesis of inflammatory responses associated with obesity mainly via the processing and release of IL‐1β.^15^, ^16^, ^17^ Several lines of evidence reported an involvement of IL‐1β in regulating the tight junction protein expression and mucosal permeability, thus contributing to the regulation of mucosal barrier integrity.^24^, ^25^ Therefore, we analyzed the release of this cytokine in cocultured EGCs (characterized by NLRP3 inflammasome activation as demonstrated above) under mimicking obesity conditions (Figure 6A).

The mixing medium (collected after 5 days of coculture) containing PA and LPS showed a significant increase in IL‐1β release, as compared with control mixing medium (Figure 6H). Of interest, when we analyzed the conditioned medium (collected 24 h after the separation of trans‐well insert) derived from cocultured IEC‐6 cells treated with PA plus LPS no significant differences in IL‐1β release were observed, as compared with related control IEC‐6 (Figure 6I). By contrast, the conditioned medium derived from cocultured activated EGCs treated with PA plus LPS displayed an increase in IL‐1β release, as compared with related control EGCs (Figure 6J), thus indicating that, in the setting of obesity, activated EGCs represent the main source of IL‐1β.

#### 2.2.4 Enteric glial NLRP3‐mediated IL‐1β release contributes to the exacerbate mucosal barrier disruption

It is known that a significant IL‐1β release can impair intestinal mucosal barrier integrity and permeability.^26^, ^27^ Therefore, we investigated the effects of the massive IL‐1β release from activated EGCs on the expression levels of tight junction proteins and trans‐epithelial permeability in intestinal epithelial cells.

**FIGURE 7:**
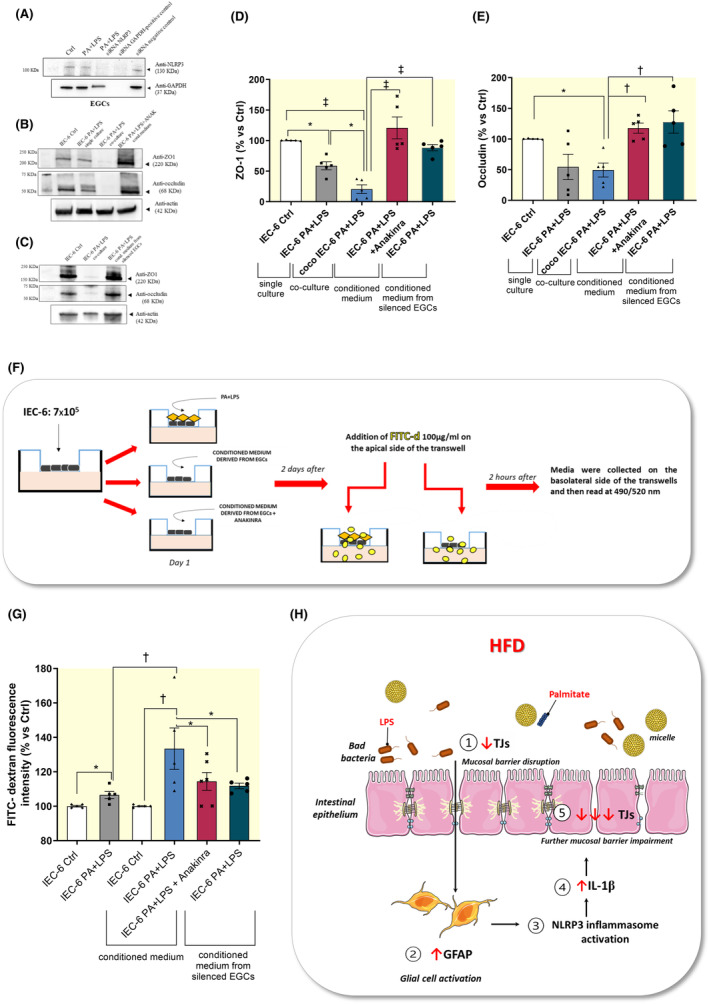
Glial NLRP3‐mediated IL‐1β contributes to further exacerbate the mucosal barrier dysfunctions associated with obesity. (A) Representative blot of NLRP3 expression assessed by Western blot assay in EGCs silenced for NLRP3 with siRNA, and subsequent incubation with PA and LPS. (B–E) A scatter plot representing the densitometric analysis and related representative blot of (B–D) ZO‐1 and (B–E) occludin assessed by Western blot assay in IEC‐6 cells incubated with conditioned medium derived from silenced‐NLRP3 EGCs or with PA plus LPS, in the absence or in the presence of anakinra, in single culture and in coculture. (F) Schematic representation of coculture experiments and FITC‐dextran experiment protocols. For experimental protocol details see “Section 4” and Figure 1B. (G) FITC‐dextran fluorescence intensity measured in IEC‐6 treated with conditioned medium derived from silenced‐NLRP3 EGCs or with PA and LPS, in the absence or in the presence of anakinra, in single culture and in coculture. Dots show values per individual experiments (*n* = 5–6 independent experiments performed in duplicate) whereas black bars indicate means ± SEM. *p < 0.05, †*p* < 0.01 and ‡*p* < 0.001. One‐way ANOVA. (H) Schematic representation of pathophysiological mechanisms underlying the impairment of IEB integrity and function associated with obesity: Interplay between intestinal epithelium and enteric glial cells in obesity. (1) HFD exposure, besides to induce alterations in IEB structure, determinates enteric gliotic processes (2) characterized by a hyperactivation of NLRP3 inflammasome (3) with consequent release of IL‐1β (4). The release of a massive levels of IL‐1β contributes to further exacerbate the disruption of mucosal barrier integrity with consequent increase of epithelial permeability (5). EGC, enteric glial cells; HFD, high fat diet; IEC‐6, intestinal epithelial cells; LPS, lipopolysaccharide; PA, palmitate; TJs, tight junctions; ZO‐1, zonulin‐1.

IEC‐6 cells (in single culture) treated with PA and LPS showed a significant reduction in the expression of ZO‐1, but not in occludin expression, as compared with related control cells (Figure 7B,D,E). Of interest, in coculture experiment the expression of ZO‐1 was further reduced along with a reduction in occludin expression in IEC‐6 cells treated with PA plus LPS, as compared with IEC‐6 cells incubated with PA plus LPS in single culture (Figure 7B,D,E). To corroborate this evidence, we performed a trans‐epithelial permeability assay (FITC assay) on IEC‐6 cells using conditioned media derived from cocultured EGCs, previously treated with PA and LPS, in the absence or in the presence of anakinra. Of note, these conditioned media contain soluble molecules (including IL‐1β) released in the medium from cells and no cell lysates. We observed that FITC‐dextran fluorescence intensity was significantly increased in IEC‐6 cells incubated with conditioned medium derived from EGCs treated with PA and LPS, as compared with IEC‐6 cells treated with PA and LPS directly in the medium (Figure 7F,G), thus highlighting the contribution of activated enteric glia in further exacerbating the disruption of mucosal barrier integrity in the setting of obesity.

Of note, in IEC‐6 cells, the incubation with IL‐1β receptor antagonist, anakinra, in the presence of the conditioned medium derived from cocultured EGCs, previously treated with PA and LPS, counteracted the reduction of ZO‐1 and occludin as well as the increase in FITC‐dextran flux (Figure 7B–G), thus highlighting the involvement of enteric glial NLRP3‐mediated IL‐1β release in the mucosal barrier dysfunctions associated with obesity. Likewise, incubation of IEC‐6 cells with conditioned medium derived from silenced‐NLRP3 EGCs counteracted the reduction of tight junction expression along with the increase of trans‐epithelial permeability (Figure 7A,C–G), thus further corroborating the contribution of glial NLRP3 inflammasome in obesity‐related intestinal barrier impairment.

## 3 DISCUSSION

The impairment of intestinal mucosal barrier has been identified as *primum movens* of a series of pathophysiologic events underlying obesity and related disorders.^4^, ^5^, ^6^ Indeed, an imbalance in the intestinal barrier structure can facilitate the translocation of bacteria and their products into the mucosa, thus promoting an uncontrollable immune reaction in the enteric microenvironment which, in turn, exacerbates the disruption of IEB, thus triggering a chronic low‐grade systemic inflammation.^7^, ^8^, ^28^ Once established, such an inflammatory background, beyond determining an enteric neuroplastic remodeling, is thought to be involved in the secretion/reabsorption alterations of the gut barrier.^29^, ^30^, ^31^, ^32^, ^33^

In this context, enteric glia exerts a pivotal role in the regulation of intestinal barrier integrity and functions as well as in the modulation of immune/inflammatory responses through the release of a plethora of pro‐inflammatory mediators.^13^, ^14^, ^34^, ^35^, ^36^ However, at present the modulatory role of enteric glia in the pathophysiology of mucosal barrier dysfunctions under inflammatory condition associated with obesity remains unclear.

Based on this background, this study was designed to elucidate the molecular mechanisms underlying the morphofunctional changes of intestinal mucosal barrier associated with obesity, paying particular attention to the interplay between intestinal epithelial cells and EGCs in the murine model of HFD‐induced obesity. We focused our attention mainly on the NLRP3 inflammasome pivotally involved in shaping immune/inflammatory responses in several disorders, including obesity.^17^ Indeed, under this pathological condition, the NLRP3 inflammasome, a key sensor of cellular stress involved in IL‐1β release, takes part actively in the immune/inflammatory responses.

The present study performed on colonic tissues from SD and HFD mice (WT and NLRP3^−/−^) as well as on in vitro coculture of EGCs with IECs, pointed out the following points of novelty: (1) NLRP3 inflammasome represents a regulatory hub linking enteric gliotic processes and intestinal mucosal barrier alterations in obesity; (2) enteric glial NLRP3‐mediated IL‐1β release contributes to the impairment of intestinal mucosal barrier associated with obesity.

In line with previous studies, we observed that HFD‐WT mice displayed a significant increase in body weight gain as well as in plasma and colonic IL‐1β levels along with a decrease of epithelial tight junction protein expression (ZO‐1 and occludin) and altered mucus production by tubular glands in colonic *tunica mucosa*, thus corroborating the existence of an interplay between HFD intake, enteric inflammation and intestinal mucosal barrier alterations.^6^, ^17^, ^37^, ^38^ In particular, HFD‐WT mice showed a significant increase in the expression of NLRP3 inflammasome subunit and active caspase‐1 in the colonic tissue, indicating the activation of the well‐established pattern of canonical inflammasome pathway.^24^ Of note, an overactivation of canonical caspase‐1‐dependent NLRP3 inflammasome pathway has been reported to contribute to the immune/inflammatory responses associated with obesity.^17^, ^38^ Indeed, several studies demonstrated that a massive increase in caspase‐1 activity and IL‐1β levels, resulting from an hyperactivation of NLRP3 inflammasome in immune cells infiltrating the white adipose tissue of obese mice, favors the onset and progression of the low‐grade inflammatory condition (also named meta‐inflammation) associated with obesity.^17^, ^38^

Interestingly, in our studies NLRP3 gene deletion in SD mice was associated with a delay in body weight gain, although not significantly, as compared with SD‐WT animals, thus suggesting an involvement of NLRP3 inflammasome in the physiological weight gain. In support to this view, a recent study demonstrated that the increase of postprandial macrophage‐derived IL‐1β induces insulin secretion with consequent increase in glucose disposal and NLRP3‐mediated inflammation.^39^ Accordingly, lack of endogenous IL‐1β signaling in mice during refeeding and obesity could reduce the concentration of insulin in plasma with consequent normalization of glucose metabolism and body weight gain.^39^ In support to this view, in our study NLRP3 gene deletion in HFD mice was associated with a comparable weight gain to that of SD‐WT mice as well as a reduction in body weight compared to HFD‐WT animals, thus corroborating the involvement of NLRP3 inflammasome in pathological weight gain associated with obesity.^17^, ^39^ In this context, no significant differences in food intake were detected in SD and HFD WT or NLRP3^−/−^ mice. In addition, HFD‐NLRP3^−/−^ mice displayed a reduction in active caspase‐1 expression as well as in circulating and colonic IL‐1β levels, thus confirming previous evidence describing the involvement of NLRP3 inflammasome in the pathophysiology of obesity and related inflammation.^17^, ^38^

It is widely recognized that the presence of a chronic low‐grade inflammatory condition in the gut induces morphofunctional changes in the cellular components of ENS with consequent alterations of intestinal motor and secretory functions.^2^, ^40^ In this context, EGCs showed to contribute actively to the onset of such bowel dysfunctions associated with obesity.^2^, ^13^, ^40^, ^41^ Under adverse conditions, EGCs acquire a pro‐inflammatory phenotype (designated as reactive gliosis),^42^ releasing a plethora of inflammatory cytokines, thus participating in the maintenance of the phlogistic process.^13^, ^43^, ^44^ An hyperactivation of NLRP3 inflammasome signaling pathway along with an increase in IL‐1β release has been reported in activated EGCs treated with LPS, confirming that enteric glial‐mediated inflammatory processes are likely to depend, at least in part, on NLRP3 inflammasome signaling pathway.^16^ Consistently with this evidence, our HFD‐WT mice, besides displaying a NLRP3 inflammasome hyperactivation and high levels of IL‐1β, showed also an increased presence of glial marker GFAP in the colonic wall. This indicates the existence of an interplay between a hypercaloric diet intake, NLRP3 inflammasome activation and the onset of a reactive gliosis process. Of note, we focused our attention on GFAP glial marker since it is modulated by cell differentiation, inflammation, and injury; therefore, its levels correlate with the functional state of EGCs, representing a useful marker for tracking glial morphologic changes associated with enteric gliotic processes.^18^, ^45^, ^46^ By contrast, other glial markers, such as Sox10 and S100‐β, represent the best markers for glial cell quantification but do not provide information on glial morphology.^18^, ^46^

In order to confirm the interplay among obesity, NLRP3 inflammasome activation and enteric gliosis, we performed a double‐staining immunofluorescence analysis to investigate ASC specks, reflecting an active status of the inflammasome,^47^, ^48^ at level of mucosal and neuromuscular GFAP‐positive glial cells. In HFD‐WT mice, an abundant number of ASC specks were observed, indicating the activation of inflammasome pathway. Of note, these results are in line with our molecular results showing an hyperactivation of NLRP3 inflammasome pathway in obese mice. In particular, an appreciable amount of ASC specks was observed in GFAP‐positive glial cells within colonic wall from HFD mice, indicating, for the first time, the activation of NLRP3 inflammasome complex in activated glial cells in paraffin tissue samples of colon. Interestingly, HFD‐NLRP3^−/−^ mice showed no increase in the NLRP3 inflammasome‐related inflammatory parameters as well as an absence of enteric gliosis, thus providing evidence, for the first time, of a link among obesity, NLRP3 inflammasome activation and reactive gliosis. Of note, it is known that phlogistic processes contribute to pathophysiological remodeling of gut barrier.^49^ Consistently, we observed that HFD‐WT mice displayed significant alterations in intestinal mucosal barrier structure. By contrast, HFD‐NLRP3^−/−^ mice (characterized by the absence of both NLRP3 inflammasome activation and mucosal enteric gliotic processes) are less susceptible to the development of mucosal barrier alterations associated with obesity, thus highlighting once again a link among HFD intake, NLRP3 inflammasome activation, enteric gliotic processes and intestinal mucosal barrier alterations.

In an attempt to clarify the potential connections/interplay among these interrelated factors, we performed a series of in vitro experiments on coculture between EGCs and intestinal epithelial cells treated with PA (the major source of fat in the hypercaloric diet) and LPS (a well‐recognized activator of the first step of NLRP3 signaling and an index of endotoxemia associated with HFD intake). Indeed, in the context of obesity, nutrients (here represented by PA) and pathogens (here represented by LPS) can translocate from the lumen to intestinal mucosa, due to intestinal barrier alterations, thus interacting with resident immune/inflammatory cells (here represented by enteric glial cells) with consequent onset of inflammatory responses.^50^, ^51^ Likewise, under our in vitro experimental condition, PA and LPS induced alterations in IEC‐6 cell structure, with a decrease in tight junction expression and an increase in FITC‐dextran flux, thus corroborating previous evidence^52^ and our in vivo data showing alterations in IEB integrity in mice fed with a hypercaloric diet. As a consequence of the impairment in IEC‐6 monolayer integrity, soluble molecules, such as PA and LPS, crossed the porous membrane of trans‐well insert resulting in an hyperactivation of beneath EGCs, as documented by an increase in GFAP expression, in accordance with previous evidence.^13^, ^14^ Of note, these results are in line with our in vivo studies showing the presence of GFAP‐positive activated enteric glial cells in obese mice.

Of interest, a recent study by Kimono et al. reported that LPS treatment determines a phenotypic pro‐inflammatory shift in EGCs, triggering an inflammatory process via the canonical NLRP3/caspase‐1/IL‐1β signaling activation.^16^ It is noteworthy that IL‐1β, beyond to be involved in the initiation/amplification of the inflammatory response, plays a critical role in the development of IEB dysfunction.^25^, ^26^, ^27^ Consistently with this evidence, our coculture experiments performed on EGCs treated with PA and LPS showed an hyperactivation of canonical NLRP3/caspase‐1 signaling pathway along with a massive IL‐1β release. This condition seems to contribute actively to a further disruption of IEC‐6 layer integrity, as documented by a decrease of tight junction molecule expression contextually with an increase in FITC‐dextran flux. Interestingly, this detrimental effect was abrogated upon the incubation of IEC‐6 cells with the IL‐1β receptor antagonist anakinra as well as with conditioned medium derived from silenced‐NLRP3 glial cells, thus confirming the contribution of enteric glial NLRP3‐mediated IL‐1β in the mucosal barrier dysfunctions associated with obesity. Of note, this in vitro evidence is in line with our in vivo results showing the absence of mucosal barrier alterations in HFD NLRP3^−/−^ mice.

Based on our findings, it is conceivable that, a hypercaloric diet intake elicits mucosal enteric gliotic processes characterized by a hyperactivation of NLRP3/caspase‐1/IL‐1β signaling pathway, that contributes to further exacerbate the disruption of intestinal mucosal barrier integrity. However, we cannot rule out the contribution of NLRP3 inflammasome activation from other cells, such as immune cells, in IEB alterations associated with obesity; therefore, focused experiments are needed to better investigate this aspect. In addition, we wish to note that this is a descriptive/correlative paper and further studies in HFD mice (WT and NLRP3^−/−^) treated with enteric glial‐selective inflammasome inhibitors (unfortunately currently not available) should be required to directly test this hypothesis, thus better substantiate the relevance of the interplay among NLRP3 inflammasome, enteric gliosis, and mucosal barrier alterations in obesity.

In conclusion, this study provides convincing evidence supporting a role of NLRP3 inflammasome complex as regulatory hub linking EGCs and intestinal epithelial cells, taking a relevant part in the pathophysiological mechanisms underlying the mucosal barrier alterations associated with obesity (Figure 7F). Based on this evidence, enteric glial NLRP3 inflammasome might represent an interesting molecular target for the development of novel pharmacological approaches aimed at managing the enteric inflammation and intestinal mucosal dysfunctions associated with obesity.

## 4 MATERIALS AND METHODS

All the material submitted conforms to good publishing practice in physiology and the *Acta Physiologica* guidelines.^53^

### 4.1 Experiments on animals

#### 4.1.1 Animals and diet

Six‐week‐old male C57BL/6 wild‐type (WT) (RRID:MGI:5658455, Envigo, Udine, Italy) and C57BL/6 NLRP3 KO (NLRP3^−/−^) (RRID: IMSR_JAX:021302) animals (kindly donated by Prof Pablo Pelegrin, Biomedical Research Institute of Murcia, Murcia, Spain) (20 g body weight) were employed during the study. Mice were fed with HFD (60% calories from fat: 36% saturated, 41% monounsaturated, 23% polyunsaturated) or standard diet (SD, 18% calories from fat: 0.9% saturated, 1.3% monounsaturated, 3.4% polyunsaturated) for 8 weeks. HFD provided 18.3% kcal as proteins, 21.4% kcal as carbohydrates and 60.3% kcal as fat, whereas SD provided 24% kcal as proteins, 18% kcal as fat and 58% kcal as carbohydrates. HFD (TD.06414) and SD (TD.2018) were purchased from ENVIGO.

The animals were housed in temperature‐controlled rooms on a 12‐h light cycle at 22–24°C and 50–60% humidity, with accessibility to food and water *ad libitum*. Body weight was measured once a week throughout the 8 weeks of SD or HFD (WT and NLRP3^−/−^). Food intake was measured and changed daily. To calculate the food intake, all remnants of pellets were weighted at 11:00, including any spilled food in cages and this value was subtracted from the initial weight. During these measurements, all mice were housed individually. After 8 weeks of diet, animals were killed by asphyxiation with CO_2_ and tissues were processed for subsequent evaluations, as described below. Of note, after 8 weeks of HFD, mice display an increase in body and epididymal fat weight, blood cholesterol, glucose, and triglycerides levels, without signs of insulin resistance.^4^, ^13^, ^17^, ^54^, ^55^ These metabolic alterations strikingly resemble those observed in obese patients not yet diabetic.^28^, ^41^, ^56^

All the procedures involving animals were carried out following the guidelines of the European Community Council Directive 86–609 and in accordance with the Code of Ethics of the World Medical Association (Declaration of Helsinki, EU Directive 2010/63/EU for animal experiments). The experiments were approved by the Ethical Committee of the University of Pisa and by the Italian Ministry of Health (authorization number 674/2016‐PR). All efforts to reduce and minimize the number of animals, and their suffering were carried out. A randomization of animals between groups was carried out in order to generate groups of equal size. The investigators responsible for data analysis were blind to which animals represent WT SD and HFD mice as well as NLRP3^−/−^ SD and HFD animals.

#### 4.1.2 Western blot assays

Colonic tissues were weighed and then homogenized in lysis buffer using a polytron homogenizer (QIAGEN, Milan, Italy), as previously reported.^57^, ^58^ Samples were then centrifuged at 12 000 rpm for 15 min at 4°C, and the resulting supernatants were separated from pellets and stored at −80°C. Bradford assay was used to quantify total proteins. Proteins (30 μg) were separated onto a precast 4%–20% polyacrylamide gel (Mini‐PROTEAN® TGX gel, Biorad) and transferred to PVDF membranes (Trans‐Blot® TurboTM, PVDF Transfer packs, Biorad). Membranes were blocked with 3% bovine serum albumin (BSA) diluted in Tris‐buffered saline (TBS, 20 mM Tris–HCl, PH 7.5, 150 mM NaCl) with 0.1% Tween 20. Primary antibodies against β‐actin (ab8227, Abcam), occludin (ab167161, Abcam), zonulin‐1 (ZO‐1, Ab96587, Abcam), GFAP (glial fibrillary acidic protein, ab53554, Abcam), nucleotide‐binding oligomerization domain leucine‐rich repeat and pyrin domain containing protein 3 (NLRP3, ab214185, Abcam) and caspase‐1 (ab1872, Abcam) were used. Secondary antibodies were obtained from Abcam (anti‐mouse ab97040 and anti‐rabbit, ab6721). Protein bands were revealed with ECL reagents (Clarity Western ECL Blotting Substrate, Biorad) and subsequently the densitometric analysis, expressed as optical density (OD), was performed with iBright Analysis software.

#### 4.1.3 Assessment of plasma and tissue interleukin‐1β levels

Plasma IL‐1β levels were measured using an enzyme‐linked immunosorbent assay (ELISA) kit (ab100704, Abcam), as described previously.^17^, ^59^ For the procedure, blood samples were centrifuged for 5 min at 4000 rpm at 2–8°C; after the centrifugation, supernatants were collected. Aliquots (100 μL) were used for the assay. IL‐1β levels were expressed as pg/mL of plasma.

IL‐1β levels in colonic tissues were measured by ELISA kit (ab100705, Abcam). Colonic tissue samples were weighed, thawed, and homogenized in 0.4 mL of PBS, pH 7.2 at 4°C, and centrifuged at 10 000*g* for 5 min. Aliquots (100 μL) of supernatants were then used for the assay. IL‐1β levels were expressed as ρg/mg of colonic tissue.

#### 4.1.4 Histological evaluations

Sections (7 μm thick) from full‐thickness distal colonic samples, formalin‐fixed and paraffin embedded, were processed for histochemical and immunofluorescence detections.

##### Histochemical detection of neutral and acidic mucins

Periodic acid‐Schiff's reagent (PAS) and Alcian Blue (AB) staining analysis was performed to quantify the neutral and acidic mucin, respectively. The neutral and acidic mucins appeared as pink and blue drops, respectively, while mixtures of neutral and acidic mucins showed purple staining.

##### Confocal immunofluorescence

Distal colonic sample sections were processed for GFAP (glial fibrillar acidic protein‐activated glial cell marker) alone or in combination with ASC (adaptor protein apoptosis‐associated speck‐like protein containing a caspase‐recruitment domain‐inflammasome activation marker) in a single and in a double immunofluorescence, respectively, as previously described.^4^, ^17^ In addition, a single immunofluorescence was performed on colonic sections to detect ZO‐1, a protein involved in tight junction complex. Briefly, sections were incubated overnight at 4°C with primary antibodies diluted in PBS: chicken anti‐GFAP (1:400, ab4674, Abcam, Cambridge, UK), rabbit anti‐ASC (1:200, AG‐25B‐006PF, Life Science, CA, USA) and rat anti‐ZO‐1 (1:200, MABT11, Merck Millipore), appropriate fluorophore‐conjugated secondary antibody, and finally with TOPRO3 for nuclear counterstaining.^4^, ^17^ Sections were examined under a Leica TCS SP8 confocal laser‐scanning microscope (Leica Microsystems, Mannheim, Germany). Since our intent was to detect the activation of inflammasome signaling in enteric glial cells from colonic mucosa of HFD mice, the results from these analyses were not subjected to statistical analyses.

### 4.2 Experiments on coculture between enteric glial cells and intestinal epithelial cells

#### 4.2.1 Cell culture

Rat‐transformed enteric glial cells (EGCs) were obtained from ATCC (EGC/PK060399egfr; ATCC CRL‐2690, Manassas, VA, USA). Cells were grown and maintained in Dulbecco's modified Eagle's medium (DMEM) supplemented with 10% fetal bovine serum (FBS), 2 mM glutamine and 100 U/mL penicillin–streptomycin in a humidified atmosphere of 5% CO_2_ at 37°C.

Rat intestinal epithelial cell line (IEC‐6) was acquired from ATCC (IEC‐6; ATCC CRL‐1592, Manassas, VA, USA). Cells were grown and maintained in DMEM plus 10% FBS, 2 mM glutamine, 100 U/mL penicillin–streptomycin and 0.1 U/mL human insulin in a humidified atmosphere of 5% CO_2_ at 37°C.

#### 4.2.2 Transwell coculture experiments

**FIGURE 8:**
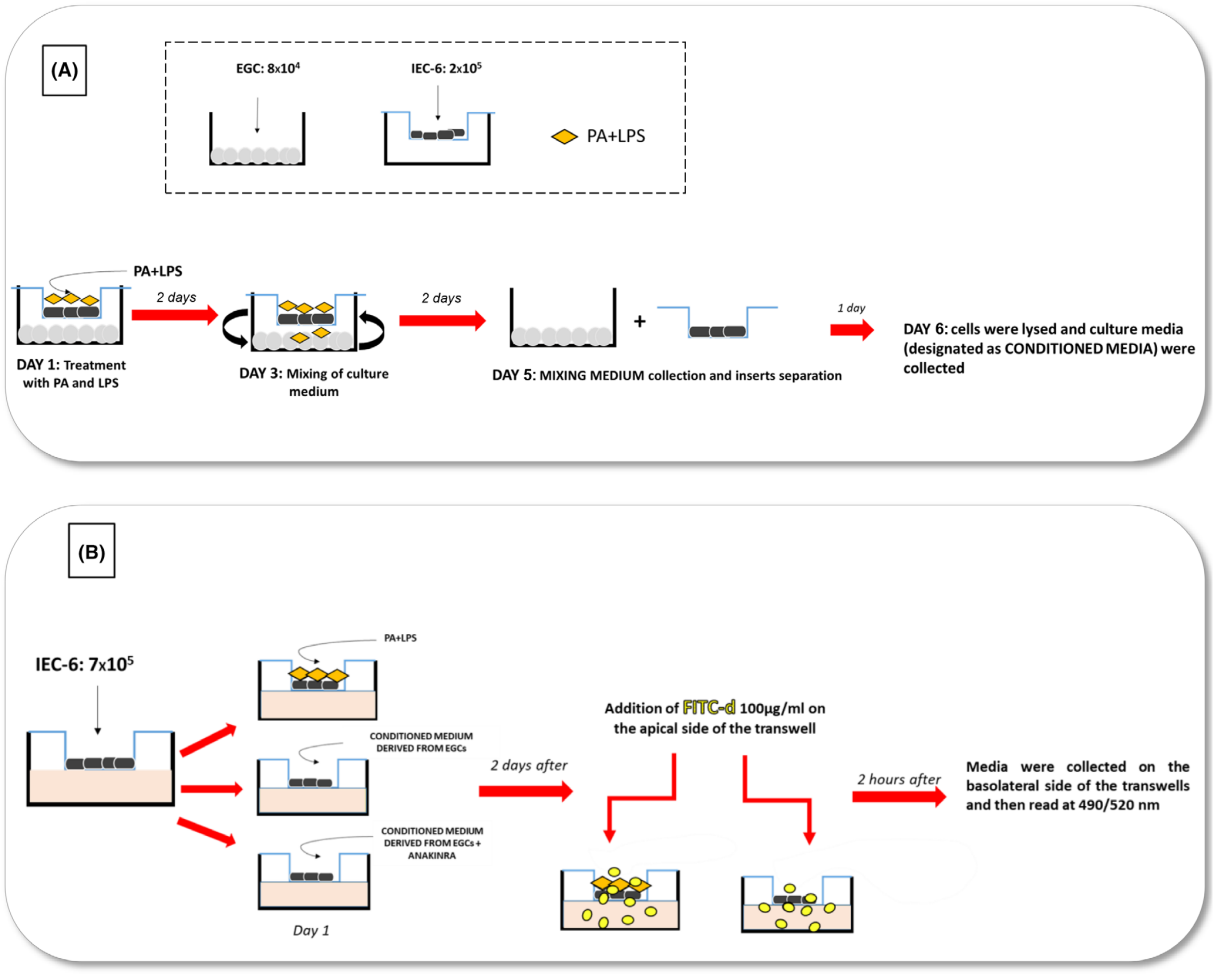
Schematic representation of coculture experiments and FITC‐dextran experiment protocols. (A) IEC‐6 cells were seeded in the apical chamber of the transwell plate. In parallel, EGCs were plated in a separate six well plates. After attachment, transwell inserts with IEC‐6 cells were transferred into wells containing EGCs. For the first 2 days, treatment with PA plus LPS was incubated in contact with IEC‐6 cells to mimic the in vivo contact of intestinal epithelium with HFD diet. After 48 h, culture media of apical and lower side of transwell plate were mixed until day 5 to allow the released soluble to interact with both cell lines. At day 5, mixing media were collected and cell lines were separated for 24 h. The next day, cells were lysed, and the soluble molecules released in the medium (designed as “conditioned medium”) have been identified and associated with the respective cell line. (B) In FITC‐dextran experiments, IEC‐6 cells were seeded in the upper side of transwell chambers on 24‐well plates. Cells were treated with: Medium containing PA plus LPS or conditioned medium derived from cocultured EGCs, previously treated with PA and LPS, in the absence or in the presence of anakinra (IL‐1β receptor antagonist). After 48 h, FITC‐dextran was added in the apical side of trans‐well insert and following 2 h of incubation, the medium in the basolateral side was collected and the absorbance of fluorescence was quantified. EGC, enteric glial cells; HFD, high fat diet; IEC‐6, intestinal epithelial cells; LPS, lipopolysaccharide; PA, palmitate.

IEC‐6 cells were seeded at a concentration of 2 × 10^5^ cells/well in the apical chamber of the transwell plate (0.4 μm pore size; Corning, Kennebunk, ME). In parallel, EGCs were plated at a concentration of 8 × 10^4^ cells/well in a separate six well plates (Figure 8A). After attachment, transwell inserts with IEC‐6 cells were transferred into wells containing EGCs and culture medium was replaced with serum‐free DMEM. IEC‐6 cells were treated with lipopolysaccharide (LPS, 10 μg/mL, Sigma‐Aldrich) and palmitate (PA, 400 μM, Sigma‐Aldrich) to mimic the in vivo features of HFD exposure (Figure 8A). Controls were run in parallel. Concentrations of PA and LPS were selected in accordance with previous reports.^13^, ^14^, ^60^, ^61^, ^62^ Of note, cells were treated with a mixture of PA and LPS based on a previous study showing that only the combination of PA and LPS was able to induce the release of pro‐inflammatory mediators and substance P from activated EGCs,^13^ thus highlighting a synergistic action of these molecules, in accordance with other studies.^63^, ^64^ For the first 2 days, treatment with PA plus LPS was incubated in contact with IEC‐6 cells to mimic the in vivo contact of intestinal epithelium with HFD diet. After 48 h, culture media of apical and lower side of transwell plate were mixed until day 5 to allow the released soluble molecules to interact with both cell lines (Figure 8A). In particular, an aliquot (250 μL) of medium from the apical was mixed with an aliquot (250 μL) of medium from the lower compartment, briefly centrifuge (to obtain a cell‐free supernatant) and then reintroduced in the upper and lower compartment. At day 5, mixing media were collected and cell lines were separated for 24 h with fresh culture media. The next day (day 6), cells were lysed and cell lysates were used for protein detection and quantification (i.e., WB); in parallel, the media (designed as “conditioned medium”) were collected and centrifuge to obtain a cell‐free supernatant and then used for the subsequent assays, including the identification and association of soluble molecules released in the medium from the respective cell line (Figure 8A). Single culture of IEC‐6 and EGCs were run in parallel.

For the experiments with IL‐1β receptor antagonist, IEC‐6 cells were treated for 48 h with 100 ng/mL anakinra (Amgen, Thousand Oaks, CA, USA) in the presence of the conditioned medium derived from cocultured EGCs, previously treated with PA and LPS.

#### 4.2.3 Palmitate solution preparation

A stock solution of PA was previously prepared as described by Voss et al.^61^ Briefly, to obtain 200 mM of stock solution, PA was dissolved in preheated water and the mixture was incubated at 70°C overnight with constant vortexing. PA‐BSA complex was prepared by mixing the stock solution to 10% bovine serum albumin (BSA, Sigma‐Aldrich). The formed complex was diluted in culture medium to prepare 400 μM PA treatment. The same final concentration of BSA (0.5% v/v) was maintained in all treated cells to avoid differential protein binding effect on compounds.

#### 4.2.4 Measurement of FITC‐dextran flux across monolayers of cultured intestinal epithelial cells

IEC‐6 cells were seeded at a concentration of 7 × 10^5^ cells/well in the upper side of transwell chambers on 24‐well plates (0.4 μm pore size; Corning, Kennebunk, ME). The next day, culture medium was replaced with medium containing PA (400 μM) plus LPS (10 μg/mL) or conditioned medium derived from cocultured EGCs, previously treated with PA and LPS, in the absence or in the presence of anakinra (Figure 8B). Controls were run in parallel. After 48 h, FITC‐dextran (FITC‐d 4 KDa, 100 μg/mL) was added in the apical side of trans‐well insert. Following 2 h of incubation, the medium (100 μL) in the basolateral side was collected (Figure 8B) and the absorbance of fluorescence was quantified using a fluorescent microplate reader (Infinite M200 pro, TECAN, Mannedorf, Switzerland) at respective excitation and emission wavelengths of 490 and 520 nm. Fluorescence levels were recorded as percentage of fluorescence versus respective control.

#### 4.2.5 Small interfering RNA (siRNA) transfection

NLRP3 siRNA transfection was performed using Lipofectamine RNAiMAX (Life Technologies, catalog 13778030) according to the manufacturer's instructions. Briefly, 1 × 10^5^ EGCs/well were transfected with targeted siRNA or scrambled siRNA (10 μM) mixed with Lipofectamine RNAiMAX diluted in optiMEM (Life Technologies, catalog #31985062) following the manufacturer's instructions. The resulting siRNA–lipid complex was added onto cells. After 24 h from siRNA transfection, EGCs were incubated with PA plus LPS for 5 days; controls were run in parallel. Knock‐down efficiency was determined by Western blotting (Figure 7A). Next, conditioned media derived from silenced EGCs were used to treat IEC‐6 cells to assessed ZO‐1 and occludin expression and FITC‐dextran flux.

#### 4.2.6 Western blot assays

Cells were lysed as previously described^65^, ^66^ and proteins were quantified using Bradford assay. Proteins were separated on a precast 4–20% polyacrylamide gel (Mini‐PROTEAN® TGX gel, Biorad) and transferred to PVDF membranes (Trans‐Blot® TurboTM, PVDF Transfer packs, Biorad). Then membranes were blocked with 3% BSA diluted in TBS (20 mM Tris–HCl, PH 7.5, 150 mM NaCl) with 0.1% Tween 20. Primary antibodies against β‐actin (ab8227, Abcam), NLRP3 (ab214185, Abcam), ASC (67824S, Cell Signaling), caspase‐1 (ab1872, Abcam), GFAP (glial fibrillary acidic protein, ab53554, Abcam), ZO‐1 (Ab96587, Abcam), and occludin (ab167161, Abcam) were used. Secondary antibodies were obtained from Abcam (anti‐mouse ab97040 and anti‐rabbit ab6721). Protein bands were detected with chemiluminescent ECL reagents (Clarityᵀᴹ Western ECL Blotting Substrate, Biorad). Then, the densitometric analysis, expressed as optical density (OD), was performed with iBright Analysis software.

#### 4.2.7 Assessment of interleukin (IL)‐1β

The release of IL‐1β was measured by ELISA kits (Abcam) into the “mixing medium” and “conditioned media” derived from EGCs and IEC‐6 cell line. Culture media were centrifuged for 5 min at 800 rpm to obtain cell‐free supernatants. Supernatants (150 μL) were then used. Absorbance was expressed in percentage versus respective control.

### 4.3 Statistical analysis

The statistical analysis of data complies with the requirements of good laboratory practices (GLP). The statistical analysis was undertaken only for studies where each group size was at least *n* = 6. In particular, the results are presented as mean ± standard error of the mean (S.E.M.). All the group sizes were designed to be homogeneous. *n* refers to the number of mice or the number of individual experiments in cell cultures, and statistical analysis was carried out using these independent values. The significance of differences was evaluated by one‐way or two‐way ANOVA followed by the appropriate post hoc test. Post hoc tests were conducted only if F in ANOVA (or equivalent) achieved a statistical significance lower than 0.05 and there was no significant variance in homogeneity. *p* values <0.05 were considered representative of significant statistical differences. All statistical procedures were performed by commercial software (GraphPad Prism, version 7.0, RRID: SCR_002798, GraphPad Software Inc., San Diego, CA, USA).

Histological data were quantitatively estimated by two blind histologists (C.S. and C.I.). PAS and AB staining in the *tunica mucosa* were acquired by a Navigator mode of Leica TCS SP8 confocal microscope (objects: 20×), equipped with a Leica DFC 7000 T camera for brightfield images (Leica Microsystems). Positive areas were estimated by the Image Analysis System “Leica Application Suite (L.A.S.) X software,” as percentage of ∑ of positive‐stained area/∑ of tissue area examined (percentage of positive pixels) and quantified as the ratio of the final value over the initial value (fold change). Data were expressed as mean ± SEM.

## AUTHOR CONTRIBUTIONS

VD, CP, and LA contributed to conception and design of the study; VD write original draft preparation; VD, LB, CP, CDS, AN, CI, and CS performed the research; VD, CP, LB, CDS, AN, CI, CS, NB, and RC collected and analyzed the data; MF, NB, RC, and LA interpreted the data; MF, NB, RC, CP, and LA coauthored the writing of the manuscript and edited the manuscript. All authors read and approved the final manuscript.

## FUNDING INFORMATION

This work was supported by the PRA 2020–2021 “Fermented cereals to reduce the obesity‐related comorbidities: preclinical and clinical evaluations” granted by the University of Pisa and PRIN 2022 (20229WP2JJ) “Brain penetrant and gut‐directed NLRP3 inhibitors in protection of physiological barriers and treatment of Alzheimer's disease (INFLA‐BAD).”

## CONFLICT OF INTEREST STATEMENT

The authors declare that the research was conducted in the absence of any commercial or financial relationships that could be construed as a potential conflict of interest.

## Data Availability

The data that support the findings of this study are available from the corresponding author upon reasonable request.
